# Vitreoretinal surgery for bilateral macular holes after laser-assisted in situ keratomileusis for the correction of myopia: a case report

**DOI:** 10.1186/1752-1947-6-381

**Published:** 2012-11-09

**Authors:** Miriam García-Fernández, Joaquín Castro-Navarro, Antonio Bajo-Fuente

**Affiliations:** 1 Department of Opthalmology, Central University Hospital of Asturias, C/Dionisio Ridruejo, nº5, 11ºD, CP: 33007, Oviedo, Spain; 2 Ophthalmologic Clinic Bajo (Vitreoretinal Surgery Department), Oviedo, Spain

**Keywords:** Laser-assisted i*n situ* keratomileusis, Macular hole, Vitrectomy

## Abstract

**Introduction:**

Laser-assisted *in situ* keratomileusis surgery may induce postoperative changes in the vitreomacular interface due to the mechanical stretch of the vitreous produced by the suction ring and the shock waves generated by the excimer laser and, subsequently, may provoke macular hole formation.

**Case presentation:**

A 53-year-old Spanish woman who had undergone a laser-assisted *in situ* keratomileusis for the correction of myopia in her right and left eye (10 years ago) was referred to our department with a complaint of decreased visual acuity in both eyes. A fundoscopy and optical coherence tomography examination revealed a bilateral full-thickness macular hole. A 23-gauge sutureless pars plana vitrectomy was performed in both eyes, and 1 month after surgery her visual acuity improved and the hole closed.

**Conclusion:**

The development of a bilateral full-thickness macular hole after laser-assisted *in situ* keratomileusis has been reported once. This case study enhances our understanding of the vitreoretinal pathology induced by laser-assisted *in situ* keratomileusis, showing the importance of a rigorous follow-up, because complications may occur even a decade later. In this case study we must also consider the contribution of the underlying myopia to the development of the bilateral macular holes.

## Introduction

There are several reports in the literature about vitreoretinal pathologic conditions after laser-assisted in situ keratomileusis (LASIK) for the correction of myopia. These include endophthalmitis [1], macular holes [2-5], retinal tears and detachments [6], retinal hemorrhages [7], and choroidal neovascular membranes [8].

Although a few papers have reported anatomic and functional outcomes after vitreoretinal surgery for the correction of unilateral full-thickness macular holes (FTMH) developing after bilateral LASIK [2-5], only one describes a patient who presented with bilateral holes after refractive laser surgery [2].

We report the results of a vitrectomy in a myopic patient who developed a bilateral FTMH after LASIK.

## Case presentation

A 53-year-old Spanish woman with myopia of −8.00 diopters in her right eye (RE) and −8.00 diopters in her left eye (LE) underwent bilateral LASIK surgery in November 2000 for her LE and February 2001 for her RE. In November 2011 she was referred to our department with a complaint of decreased vision in both eyes for approximately 4 months.

At the first visit, her best corrected visual acuity (BCVA) was 0.4 in RE and 0.2 in LE.

**Figure 1:**
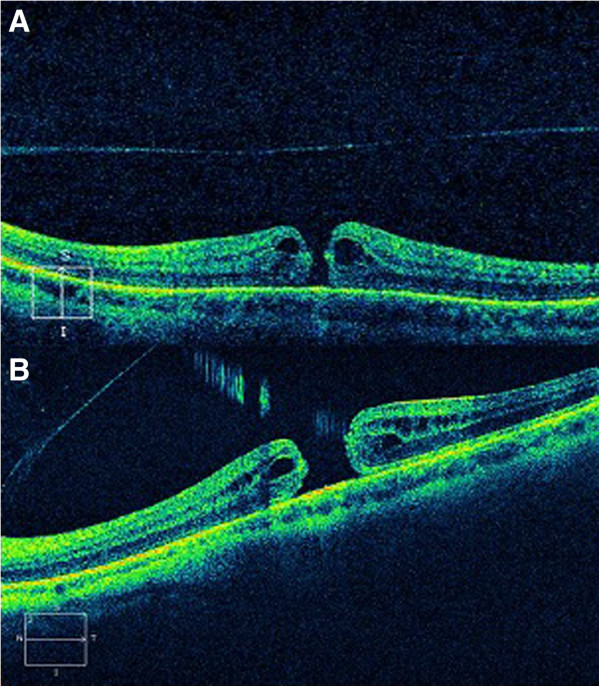
**Optical coherence tomography (cirrus spectral domain) at first visit, revealing a full-thickness macular hole in both eyes. A**: right eye. **B**: left eye.

An anterior segment examination revealed a post-LASIK cornea with no further abnormalities in both eyes. A fundoscopy revealed an image of FTMH in both eyes. An optical coherence tomography (OCT) (cirrus spectral domain; Carl Zeiss Meditec, Germany) examination showed a stage IV macular hole in RE and LE, with subretinal fluid surrounding the defect, and an absence of yellow deposits on the retinal pigment epithelium (Figure 1). We also observed a posterior vitreous detachment in both eyes.

A 23-gauge sutureless pars plana vitrectomy (PPV) and sulphur hexafluoride gas as tamponade was performed at 30 days after diagnosis with a time interval of 1 month between both eyes. A prone position was advised for 1 week.

**Figure 2:**
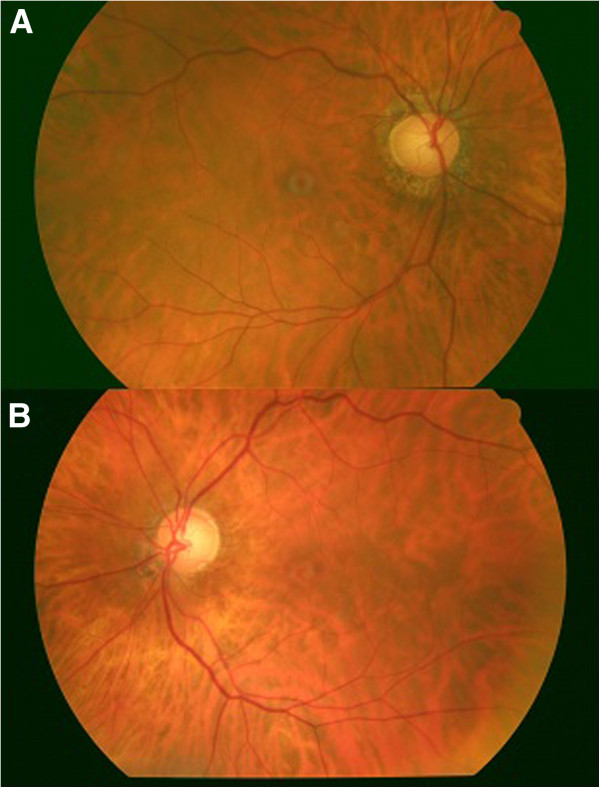
**Fundoscopic appearance at 1 month after vitrectomy. A**: right eye. **B**: left eye.

**Figure 3:**
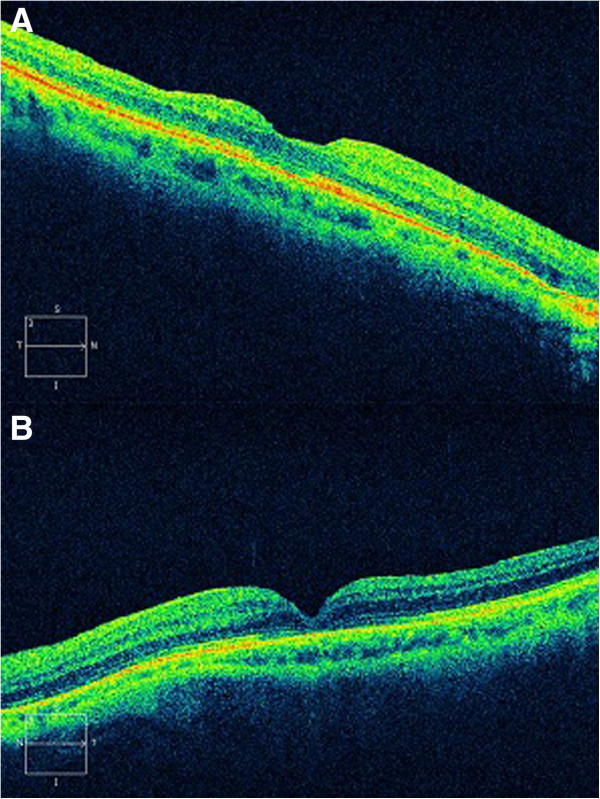
**Optical coherence tomography (cirrus spectral domain) at 1 month after vitrectomy showing the closure of the macular hole in both eyes. A**: right eye. **B**: left eye.

One month after vitrectomy, the patient’s BCVA was 1.0 in RE and 0.6 in LE. A retinal examination revealed the closure of the macular hole in both eyes (Figure 2), and OCT confirmed the restoration of the macular defect (Figure 3). However, we could appreciate a slight decrease in the foveal central thickness (125μ of residual foveal thickness) of the patient’s LE. We also observed a moderate alteration of the junctional layer of the inner and outer segments of photoreceptors in both eyes.

Six months after surgery the visual acuity and the anatomic appearance remained unchanged.

## Discussion

Nowadays, LASIK is one of the most popular alternatives for correction of low and moderate myopia. Serious complications after LASIK are infrequent and vitreoretinal pathologic conditions, if managed promptly and adequately, can result in good vision [9].

It is well known that myopia is a risk factor for macular hole formation and changes in vitreomacular interface are involved in its pathogenesis.

LASIK surgery may induce postoperative changes in this interface, for instance, the mechanical stretch of the vitreous produced by the suction ring, might subsequently provoke macular hole formation.

Nevertheless, it is not an easy task to determine if these are myopic holes that would have developed regardless of LASIK [2].

Several cases of unilateral macular hole after LASIK have been described in the past decade [2-5].

Ruiz-Moreno *et al*. [4] described the case of a patient who developed a unilateral macular hole 12 months after bilateral LASIK for correction of a moderate myopia. Prior to vitreoretinal surgery the patient’s BCVA was 20/100, and after PPV with internal-limiting membrane peeling the patient’s BCVA improved to 20/50 [4].

Chan and Lawrence [5] reported that three myopic patients developed a macular hole in one eye after bilateral LASIK or photorefractive keratectomy. A vitrectomy closed the macular hole of two cases with a final best-corrected visual acuity of 20/25 in Case 1 and 20/30 in Case 2, whereas the other patient declined further surgery.

Arevalo *et al*. [3] reported the largest series of patients with FTMH after LASIK. They analyzed 14 eyes of 13 patients with macular hole development a mean of 13 months after refractive surgery. Posterior vitreous detachment was observed in 42.8% of the eyes after LASIK, and it was not present before. The macular hole was unilateral in 12 of 13 patients (although one of those patients had an impending macular hole in the fellow eye). Surgery closed the macular hole on all 14 eyes, and functional recuperation occurred in 13 of 14 eyes (92.8%).

Thus, all these papers showed that vitreoretinal surgery can be useful in restoring vision for most myopic eyes with FTMH after refractive laser surgery. However, only one of the 24 patients described until now with FTMH development after LASIK showed bilateral presentation as happened in our case study.

Our case showed an extremely infrequent situation, as there is only one previous report describing bilateral full-thickness macular appearance after LASIK. It is well known that patients with bilateral high myopia who have FTMH and retinal detachment in one eye are expected to be at increased risk of retinal detachment in the other eye.

We postulate that in the present case study an identical refraction in both eyes (−8 diopters), and thus a similar grade of degeneration of retinal tissue, may have contributed to a similar response to the same amount of damage induced by LASIK. However, the cases previously reported in the literature were probably patients with a different refractive sphere between both eyes, which consequently required a different intensity of the shock waves generated by the excimer laser, and this caused different grades of retinal damage, such as a macular hole in one eye but not in the other eye.

We must also consider the possibility of macular hole formation secondary to myopic changes, without the influence of LASIK.

An important limitation of our case report is that OCT was not performed before LASIK, and we do not know exactly the appearance of the vitreomacular interface and, subsequently, we cannot determine precisely if the posterior hyaloid was detached or not prior to laser refractive surgery.

## Conclusion

To the best of our knowledge this is the second case report in the literature which describes a bilateral macular hole formation after myopic LASIK.

In our case study the most feasible explanation is that both factors (high myopia and LASIK) have contributed to the macular pathology: the LASIK surgery accelerating the occurrence of the posterior vitreous detachment, which was responsible for the formation of a bilateral macular hole in the eyes; and an anatomic predisposition due to the underlying high myopia.

### Consent

Written consent was obtained from the patient for publication of this case report and any accompanying images. A copy of the written consent is available for review by the Editor-in-Chief of this journal.

## Competing interests

The authors declare that they have no competing interests.

## Authors’ contributions

MGF was involved in data acquisition and manuscript drafting. JCN and ABF were involved in data interpretation and revised the report critically for important intellectual content. All authors read and approved the final manuscript.
